# Alginate oligosaccharides enhance diffusion and activity of colistin in a mucin-rich environment

**DOI:** 10.1038/s41598-022-08927-1

**Published:** 2022-03-23

**Authors:** Joana Stokniene, Mathieu Varache, Philip D. Rye, Katja E. Hill, David W. Thomas, Elaine L. Ferguson

**Affiliations:** 1 Advanced Therapies Group, School of Dentistry, College of Biomedical and Life Sciences, Cardiff University, Cardiff, CF14 4XY UK; 2 AlgiPharma AS, 1337 Sandvika, Norway; 3 Present Address: Advanced BioDesign, Parc Technologique de Lyon/Bâtiment D, 655 Allée des Parcs, 69800 Saint-Priest, France

**Keywords:** Microbiology, Antimicrobials, Biofilms, Bacterial infection

## Abstract

In a number of chronic respiratory diseases e.g. cystic fibrosis (CF) and chronic obstructive pulmonary disease (COPD), the production of viscous mucin reduces pulmonary function and represents an effective barrier to diffusion of inhaled therapies e.g. antibiotics. Here, a 2-compartment Transwell model was developed to study impaired diffusion of the antibiotic colistin across an artificial sputum (AS) matrix/medium and to quantify its antimicrobial activity against *Pseudomonas aeruginosa* NH57388A biofilms (alone and in combination with mucolytic therapy). High-performance liquid chromatography coupled with fluorescence detection (HPLC-FLD) revealed that the presence of AS medium significantly reduced the rate of colistin diffusion (> 85% at 48 h; *p* < 0.05). Addition of alginate oligosaccharide (OligoG CF-5/20) significantly improved colistin diffusion by 3.7 times through mucin-rich AS medium (at 48 h; *p* < 0.05). Increased diffusion of colistin with OligoG CF-5/20 was shown (using confocal laser scanning microscopy and COMSTAT image analysis) to be associated with significantly increased bacterial killing (*p* < 0.05). These data support the use of this model to study drug and small molecule delivery across clinically-relevant diffusion barriers*.* The findings indicate the significant loss of colistin and reduced effectiveness that occurs with mucin binding, and support the use of mucolytics to improve antimicrobial efficacy and lower antibiotic exposure.

## Introduction

Over the past three decades, mortality due to chronic respiratory diseases has increased by 18%, accounting for 7% of all deaths worldwide^1^. Respiratory mucins secreted by surface epithelial goblet cells and mucous cells of submucosal glands, play an important role in protecting the lung from environmental factors, but conversely their inflammation-associated hypersecretion contributes to pathogenesis in muco-obstructive diseases, e.g. cystic fibrosis (CF) and chronic obstructive pulmonary disease (COPD)^2^. Impaired mucociliary clearance results in a cycle of chronic bacterial infection, airway inflammation and obstruction, leading to respiratory failure and increased mortality in CF patients^3^. Within the mucin-rich CF lung, bacteria are embedded in a complex, charged, hydrated extracellular polymer matrix (EPS). The precise EPS composition varies depending on bacterial species and physicochemical environment; being comprised of host- (e.g. extracellular [e]DNA) and bacterial-derived extracellular polysaccharides (e.g. alginates)^4^. This polymer network confers considerable fitness advantages to bacteria in resisting hydrodynamic shear and, importantly, chemical disruption via systemic and inhaled antimicrobial therapies^5–7^. In chronic respiratory disease, it is evident that diffusion of therapeutic agents across the biofilm and mucus barrier may be impaired via charge-interactions^8,9^, and by binding to specific components of the entangled mucin polymer network^10^.

The antibiotic colistin has emerged as an antibiotic of ‘last resort’ against multidrug-resistant (MDR) Gram-negative bacteria and is increasingly used as an inhalation therapy to treat chronic airway infections caused by *Pseudomonas aeruginosa* in patients with CF^11,12^. Although the prevalence of *P. aeruginosa* infections has decreased in individuals with CF over the last 15 years, it still remains the predominant pathogen, affecting ~ 80% of adults^13^. Colistin is a cationic amphipathic antibiotic composed of at least 30 closely related molecules, with colistin A (polymyxin E1) and B (polymyxin E2) being the major constituents^14^. Colistin bactericidal activity is driven by electrostatic interactions between the antibiotic cationic amino groups and lipopolysaccharide (LPS) anionic phosphate groups on the outer membrane of Gram-negative bacteria. These displace magnesium and calcium cations, disrupting the physical integrity of the bacterial outer membrane, causing cell death^15^. Despite potent in vitro antimicrobial activity against *P. aeruginosa*^16,17^, colistin effectively binds to mucin in CF sputum or on the airway epithelium, substantially reducing its availability and efficacy^10^.

To increase the effectiveness of antibiotic delivery across the mucosal barrier, a number of therapeutic approaches have been described that disrupt the structural integrity of the mucin polymer matrix, including hypertonic saline and gentamicin^18^, *N*-acetyl cysteine (NAC) and clarithromycin^19^, and latterly poly(ethylene glycol)-co-poly(D,L,-lactide-co-glycolide) diblock (PEG-PLGA) nanoparticles with tobramycin^20^.

OligoG CF-5/20 (OligoG) is a low molecular weight alginate oligosaccharide (Mn 3200 g/mol) derived from the stem of brown algae *Laminaria hyperborea,* currently undergoing clinical trials as an inhalation therapy in CF patients. OligoG has previously been shown to modify the surface charge (at pH 5 and 7) and porosity of the respiratory mucin matrix^21–23^, potentiate the efficacy of antibiotics against MDR pathogens^24^ and disrupt the EPS of bacterial biofilms^25^. Disruption of the cross-linked respiratory mucin polymer network in CF mucus could greatly improve the bioavailability, diffusion and efficacy of inhaled antibiotics at the site of infection^26^.

Several strategies have been employed to analyze mucin-drug interactions in vitro^27^, including the use of microfluidic mucus-chips^28^, UV–visible localized spectroscopy^29^ and the Transwell diffusion model^30^. We have previously demonstrated ability of OligoG to bind mucin and alter the viscoelastic properties of CF sputum^21,22^. Therefore, this study aimed to investigate the ability of OligoG to improve mucus penetration by colistin, to enhance its antimicrobial activity against mucoid *P. aeruginosa* biofilms using a Transwell diffusion model.

## Results

### Colistin quantification in the lower Transwell compartment using high-performance liquid chromatography-fluorescence detection (HPLC-FLD)

**Figure 1:**
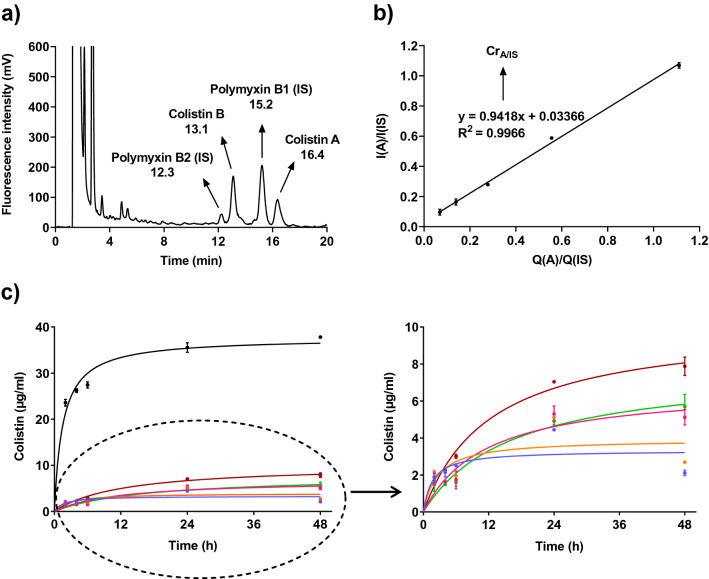
Optimization of colistin detection and quantification using high-performance liquid chromatography-fluorescence detection. (**a**) A typical chromatogram showing colistin detection in a polymyxin B spiked sample following colistin diffusion through artificial sputum (AS) medium. (**b**) Calibration curve of colistin. (**c**) Colistin quantification in the lower Transwell compartment after diffusion through AS medium ± OligoG at 0.5, 1 and 2% added to the surface of AS medium or pre-incubated for 4 h (± SD; n = 3). Abbreviations: PBS (black), AS (blue), 1% OligoG (surface treatment; orange), 0.5% OligoG (4 h pre-incubation; pink), 1% OligoG (4 h pre-incubation; green), 2% OligoG (4 h pre-incubation; burgundy).

Initial experiments sought to optimise quantification of colistin in the model. A typical HPLC chromatogram of a Transwell diffusion sample spiked with the internal standard (IS), polymyxin B, is shown in Fig. 1a. A peak corresponding to colistin was observed in the sample from the lower Transwell compartment, which was separate to that of the IS (polymyxin B) and was clearly distinguishable from artificial sputum (AS) medium impurities or derivatization by-products (results not shown). Separation of polymyxin species was achieved within 20 min; the FMOC derivatives of colistin B and A eluting at 13.1 and 16.4 min and polymyxin B2 and B1 at 12.3 and 15.2 min, respectively. The colistin calibration curve is shown in Fig. 1b.

Initial diffusion of colistin in PBS through the Transwell membrane increased over time and reached 94.5% of the initial applied dose at 48 h (Fig. 1c and Table 1). In contrast, a substantial reduction in colistin diffusion through AS medium was evident (> 85% at 48 h; *p* < 0.05), with only 5.6% of the initial applied dose detected in the lower compartment after 48 h. Pre-treatment of AS medium with OligoG (at 0.5, 1 or 2%) caused a concentration-dependent increase in the amount of colistin detected in the lower Transwell compartment, changed the slope and improved the diffusion rate (up to 3.7 times), reaching 13.6, 15.1 and 20.8% (Table 1) of the applied colistin dose after 48 h, respectively (*p* < 0.05). Surface treatment with 1% OligoG proved less effective than pre-treatment, leading to detection of only 7.1% of the applied colistin dose in the lower chamber after 48 h.

**Table 1:** Percentage of the original colistin dose detected in the lower Transwell compartment after 48 h diffusion through artificial sputum (AS) medium ± OligoG using a high-performance liquid chromatography-fluorescence detection method. Significant difference is indicated by *, where *p < 0.05 compared to OligoG untreated control.

Artificial sputum treatment	% of colistin original dose				
	2 h	4 h	6 h	24 h	48 h
PBS (no AS medium)	59.0	65.5	68.6	88.9	94.5
No OligoG	7.9	8.2	9.2	12.5	5.6
1% OligoG (surface treatment)	9.3	8.2	7.1	14.4	7.1
0.5% OligoG (4 h pre-incubation)	6.6	6.3	6.3*	14.9*	13.6*
1% OligoG (4 h pre-incubation)	6.3	5.7	5.9*	13.8	15.1*
2% OligoG (4 h pre-incubation)	8.0	8.6	11.0	19.8*	20.8*

### Metabolic activity of *P. aeruginosa* NH57388A in the Transwell diffusion model

**Figure 2:**
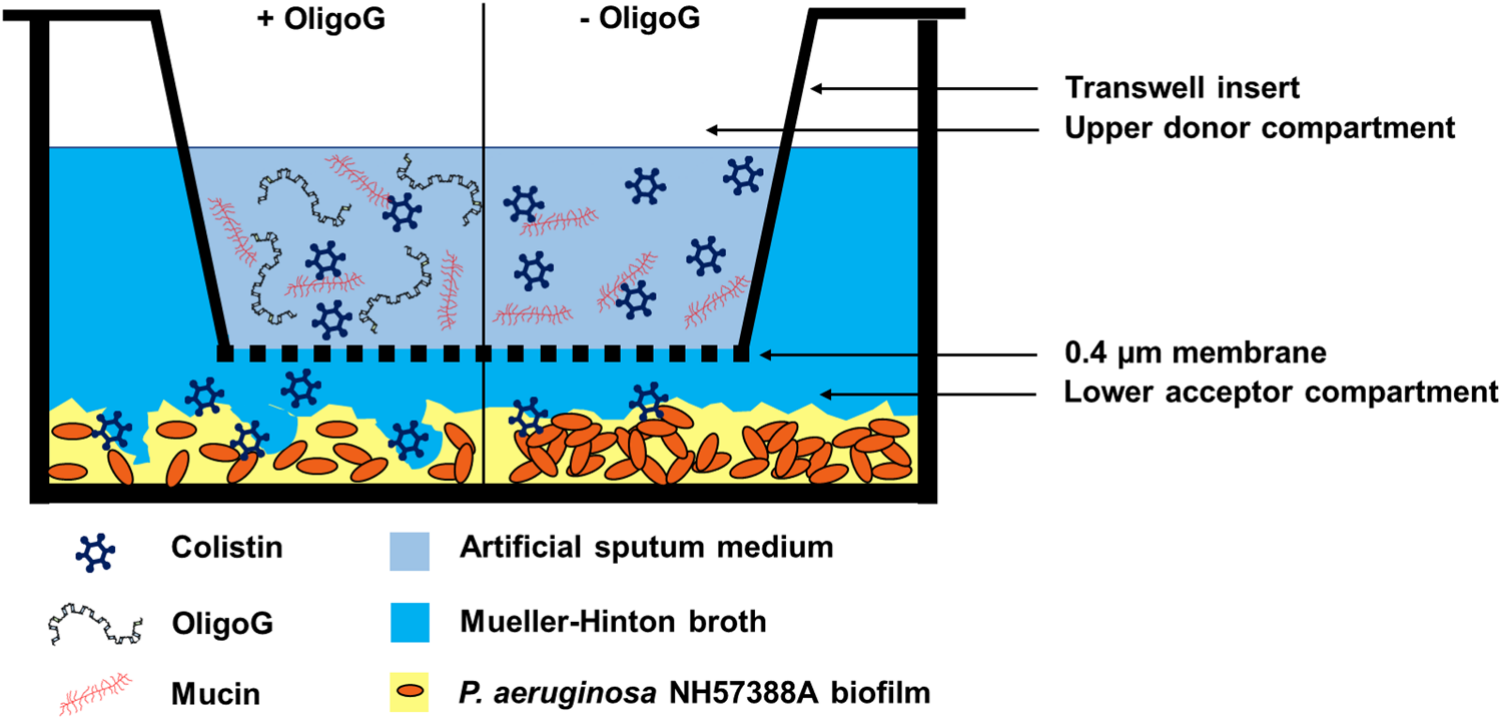
Schematic diagram of the Transwell diffusion model indicating colistin diffusion through artificial sputum medium ± OligoG and antimicrobial activity against cystic fibrosis clinical isolate *P. aeruginosa* NH57388A.

**Figure 3:**
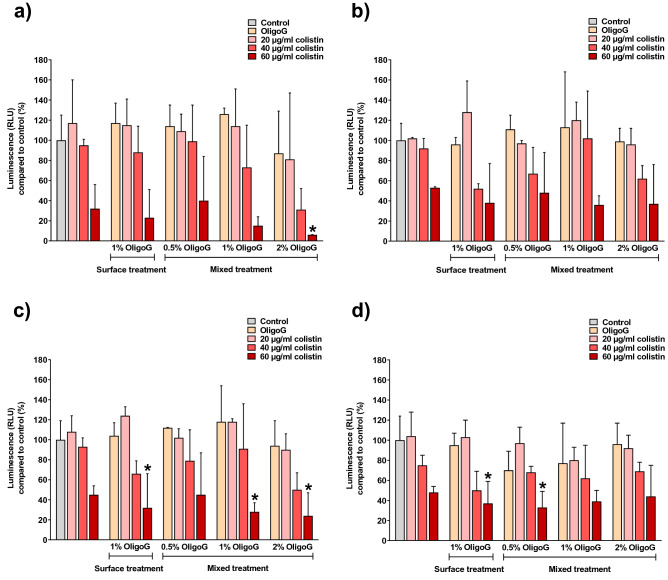
Cell viability (showing ATP production using the CellTiter-Glo assay) of 24 h *P. aeruginosa* NH57388A biofilms, following 24 h treatment with colistin ± OligoG (0.5, 1 and 2% w/v) added either directly to the artificial sputum (AS) medium surface or pre-incubated with AS medium for 4 h (± SD; n = 2). (**a**) Planktonic bacteria from the biofilm supernatant. (**b**) Planktonic bacteria after biofilm washing. (**c**) Total planktonic bacteria (a + b). (**d**) Attached biofilm bacterial population. Significant difference is indicated by *, where *p < 0.05 compared to untreated control.

A representative schematic of the Transwell diffusion model is presented in Fig. 2. To assess effective diffusion of colistin through the model, an ATP cell viability assay was used to quantify metabolically active (viable) *P. aeruginosa* NH57388A cells in the lower chamber following treatment. It was evident from the results that viability of planktonic bacteria decreased with increasing concentration of OligoG, reflecting the increased colistin diffusion to the lower Transwell compartment (Fig. 3a–c). Although ≥ 40 µg/ml colistin caused a decrease in *P. aeruginosa* NH57388A cell viability, only 60 µg/ml colistin with 1 or 2% OligoG (surface treatment or pre-incubation for 4 h) caused a significant reduction in ATP production, compared to the untreated control (Fig. 3c; *p* < 0.05). A similar trend was observed for the attached biofilm cell population, however the cell viability decrease was only significant (Fig. 3d; *p* < 0.05) at 60 µg/ml colistin with 0.5 or 1% OligoG (surface treatment or pre-incubation for 4 h).

### Antimicrobial activity of colistin against *P. aeruginosa* NH57388A biofilms

**Figure 4:**
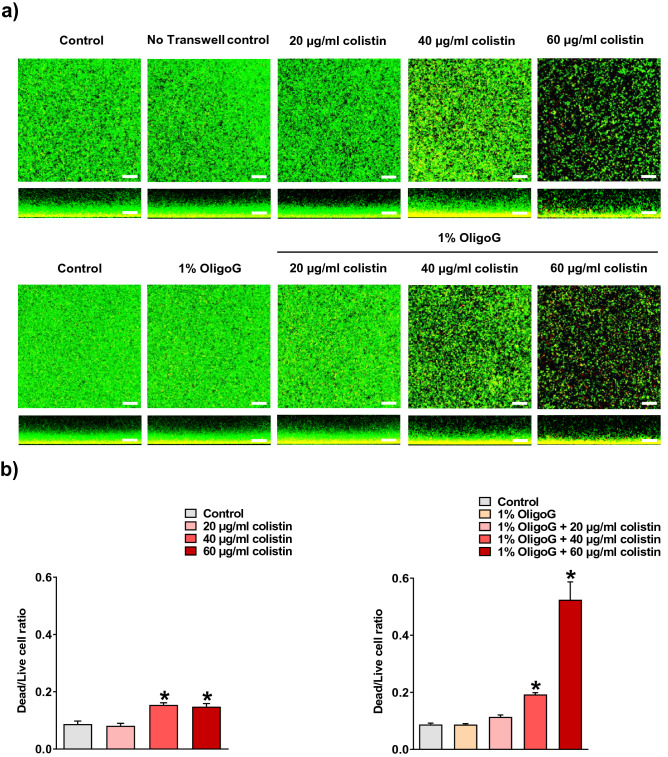
(**a**) Biofilm disruption assay in Transwell plates showing LIVE/DEAD (green/red, respectively) stained confocal laser scanning microscopy (CLSM) images (aerial and cross sectional views, scale bar, 30 μm) of 24 h *P. aeruginosa* NH57388A biofilms, following 24 h treatment with colistin ± 1% OligoG added onto the artificial sputum medium surface. (**b**) COMSTAT image analysis of the CLSM images (± SEM; n = 15). Significant difference is indicated by *, where *p < 0.05 compared to untreated control.

Effective diffusion of colistin through the model was also corroborated by analysis of direct antimicrobial effects on the bacterial biofilm in the lower chamber. Confocal laser scanning microscopy (CLSM) of LIVE/DEAD stained *P. aeruginosa* NH57388A biofilms grown in the lower chamber of the Transwell model for 48 h demonstrated well-defined homogeneous growth in the untreated control ± Transwell insert (Fig. 4a). CLSM images and COMSTAT analysis showed a concentration-dependent biofilm disruption, following 24 h treatment with colistin ± 1% OligoG. Although colistin alone (40 and 60 µg/ml) significantly disrupted the *P. aeruginosa* NH57388A biofilm structure, addition of 1% OligoG on the colistin-containing AS medium surface, caused a substantial reduction of biofilm thickness (observed in the cross-sectional views of CLSM images) that was associated with a significantly larger increase (*p* < 0.05) in bacterial cell death, compared to untreated control (Fig. 4b).

### Analysis of microbial growth of *P. aeruginosa* NH57388A in the Transwell diffusion model

**Figure 5:**
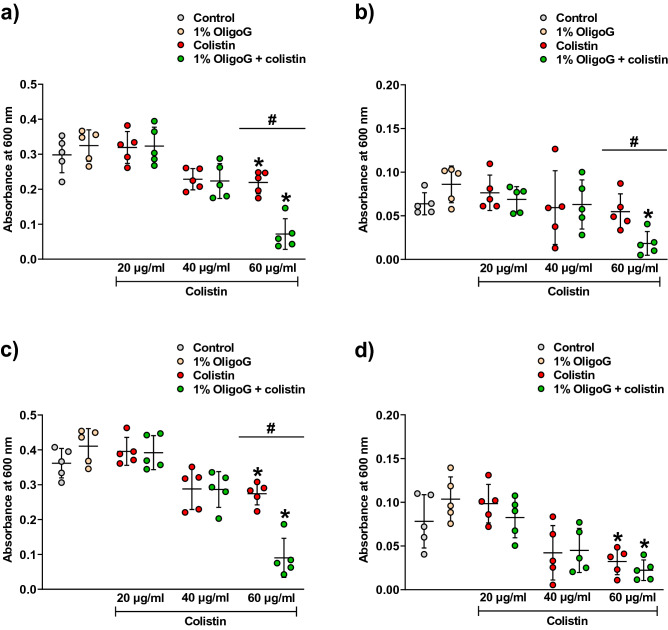
Analysis of planktonic (supernatant) and biofilm growing cells of 24 h *P. aeruginosa* NH57388A, following 24 h treatment with colistin ± 1% OligoG added onto the artificial sputum medium surface (± SD; n = 5). (**a**) Planktonic bacteria from the biofilm supernatant. (**b**) Planktonic bacteria after biofilm washing. (**c**) Total planktonic bacteria (a + b). (**d**) Attached biofilm bacterial population. Significant difference is indicated by *, where *p < 0.05 compared to untreated control and ^#^*p* < 0.05 between two groups as indicated by horizontal bar.

Effective diffusion of colistin through the model was also confirmed by direct effects on bacterial cell density (growth) in the lower chamber. Antibacterial activity was concentration-dependent with a significant decrease (*p* < 0.05) in bacterial cell density (both planktonic and biofilm; Fig. 5) demonstrated at 60 µg/ml colistin, which was augmented by the addition of OligoG (*p* < 0.05). At colistin concentrations (≥ 40 µg/ml) ± 1% OligoG (surface treatment) a marked reduction in bacterial cell density (both planktonic and biofilm cells) was observed, compared to the untreated control, however, this was not significant for any of the treatment groups. Colistin at 20 µg/ml ± 1% OligoG, or OligoG alone, had no apparent effect on bacterial growth.

## Discussion

In the CF airway, biofilms are embedded within the complex mucin-rich respiratory mucus that represents an additional challenge for antibiotic delivery via inhalation therapy. Previous studies have shown that respiratory mucins can alter the structural assembly and growth of bacterial biofilms in this environment, affecting both susceptibility and tolerance to antibiotic treatments^5,31^. Here, the composition of AS medium was adapted from previous studies^32,33^ and modified to represent a clinically-relevant diffusion model of the CF lung. Although the use of CF patient sputum in the Transwell diffusion model would be considered ideal, it is difficult to standardize due to its high inherent variability in composition and viscoelastic properties, while the presence of antibiotics and other therapeutics would present additional challenges in data interpretation^22^. The optimal concentration of mucin to use in AS medium to mimic the composition of the CF lung is unclear; previous studies have used a wide range including 5 mg/ml^32^, 10 mg/ml^34^, 20 mg/ml^35^, 30 mg/ml^36^ and 40 mg/ml^37–39^. In a clinical setting, a dramatic increase in mucin levels during pulmonary exacerbations has been reported in CF patients^40^ and therefore, in this study, a higher content of mucin (40 mg/ml) was chosen to study colistin diffusion.

As expected, colistin diffusion through AS medium was highly impaired as only 5.6% of the original dose was detected in the lower Transwell compartment after 48 h. The in vivo pore diameter of the CF sputum network has been reported to range from 0.1 to 0.4 µm^41^, which might limit the penetration of antibiotics. Accordingly, Transwell inserts with a 0.4 µm pore size were selected to provide a clinically-relevant permeability barrier while preventing leakage of AS components through the pores. Due to interactions with respiratory mucins^21^, OligoG was predicted to remain predominantly within the three-dimensional mucin network on the top of the membrane in the upper Transwell well, which proved to be the case.

Colistin diffusion in PBS through AS medium was practically unimpeded, with almost 95% of the initial dose detected in the lower Transwell compartment. In contrast, permeation of colistin in AS medium was limited by mucin entanglement, likely due to colistin-binding to specific components of the AS medium, depending on mucin content and charge interactions. Indeed, the efficacy of nanoparticle diffusion through CF sputum has been shown to be strongly influenced by mucin concentration, being significantly reduced at 25, 30 and 50 mg/ml (to ~ 82%, ~ 72% and ~ 55% respectively), with no effect observed with mucin ≤ 10 mg/ml^36^. A significant inhibition in diffusion rate of β-lactam antibiotics through mucin (40 mg/ml) has also been demonstrated^42^. Furthermore, at very high concentrations of porcine mucin (125 mg/ml), extremely low levels of free polymyxin antibiotics (15% of colistin and 16% of polymyxin B) have been reported^10^, which further supports the results observed in this study and suggests strong interactions between mucins and colistin.

Mucin concentration in CF sputum is reported to vary between patients, from 8 to 47 mg/ml^43^ and is highly dependent on mucus concentration (% of solids)^44^. The high variability observed in pre-clinical planktonic assay systems may in part, explain the often, poor responses observed in human studies and problems with antibiotic dosing in vivo. Previous studies have shown the ability of OligoG to significantly modify the three-dimensional mucin network in CF sputum by inducing morphological changes and increasing porosity within the mucin structure in a dose-dependent manner^22^. This was also evident in the present study, as pre-treatment of AS medium with increasing concentrations of OligoG caused markedly higher and sustained diffusion of colistin over 48 h, reaching up to 21% of the initial applied dose. OligoG also binds to mucins, altering its surface charge^22^. Since the charge distribution of the mucin network has been shown to be critically important for diffusion through mucin barriers^8^, this may also explain the observed increase in colistin diffusion in OligoG-treated AS medium.

Inhaled antimicrobials such as colistin, tobramycin and aztreonam lysine are the most commonly used antibiotics for the treatment of chronic *P. aeruginosa* infection in CF patients^45^. Inhalation therapy offers many advantages over systemically administered drugs, as high drug concentrations can be delivered directly to the airways, thereby improving efficacy and minimising systemic absorption and toxicity. This is especially important for antibiotics such as the polymyxins, as concentration-dependent nephrotoxicity has been reported after systemic administration^46^ which can compromise effective dosing in CF patients. Whilst colistin concentration in epithelial lining fluid may vary substantially (9.5–1137 µg/ml) between critically ill patients^47^, the colistin concentration range employed here (20–60 µg/ml) was intended to reflect current antibiotic dosing delivered via inhalation^16,48^. Substantial (concentration-dependent) biofilm disruption was noted following treatment with the colistin and OligoG combination in this model. Previously, OligoG has been shown to potentiate the efficacy of colistin against *P. aeruginosa* biofilms in vitro and in vivo but had no apparent effect on the planktonic growth^49^. As expected, addition of OligoG induced greater colistin diffusion, that correlated with a significantly higher biofilm Dead/Live cell ratio. Poor penetration of fluorescently-labelled colistin (12–19% of initial dose) into the *P. aeruginosa* biofilm matrix in an ex vivo porcine lung model has also been reported^50^, suggesting that high doses of antibiotic are required to combat chronic pulmonary infections. The maximum reported sputum concentration for inhaled colistin is ~ 40 µg/ml^16^. Although the highest dose tested here (60 µg/ml colistin) could not completely eradicate *P. aeruginosa* biofilms, combination with OligoG substantially improved the efficacy of colistin, as confirmed by the significantly lower bacterial cell densities and reduced cell viability (ATP production).

Previous studies have also shown concentration-dependent binding of tobramycin to mucin and DNA. It was suggested that a change in dosing regimen from twice daily to taking the combined dose once a day could overcome problems associated with antibiotic binding to these components of CF sputum and may result in more effective treatment of highly resistant *P. aeruginosa* strains^51^. This has proved to be the case in practice, with increased tobramycin dosing leading to higher levels of free drug detected in CF sputum^52^. Similar changes in dosing might also be effective for colistin, as diffusion is markedly impeded by the mucin barrier, allowing substantially higher peak sputum levels to be achieved. Combination therapy with OligoG may further potentiate the efficacy of colistin.

Colistin exhibits poor UV absorbance and is not inherently fluorescent, so its quantification in biological fluids can be extremely challenging. Here, detection of colistin in the lower Transwell compartment was performed using HPLC-FLD with colistin derivatization to a fluorescent derivative^53^ and inclusion of an internal standard^54^ for increased sensitivity and accuracy. The intra-day and inter-day precisions for colistin A and colistin B have been reported to be below 9.9% and 4.5% relative standard deviations, respectively and accuracy between 100.2 and 118.4%^54^. Polymyxin B was chosen as the internal standard due to its structural similarity to colistin and the presence of amino groups suitable for efficient derivatization. Indeed, no interference or peak overlap was observed, allowing a clear separation of colistin (colistin B and A), polymyxin B (polymyxin B2 and B1) and sample impurities. Thus, HPLC-FLD provided a reliable method for colistin detection and quantification in complex culture medium.

Despite the reproducible results presented here, it should be noted that optimization of the Transwell model system and colistin quantification in the lower well took considerable effort. A variety of other techniques have been employed to characterise the mucin polymer matrix in CF sputum, including atomic force, scanning electron or confocal imaging microscopy^55^, fluorescence recovery after photobleaching^56^, rheology (to measure viscoelastic properties)^57^ and multiple particle tracking (MPT; to assess the microstructure of mucus and matrix pore size using nanoparticle diffusion)^58^. Recently, MPT was used to characterize the mechanical robustness of Gram-negative and -positive bacterial biofilms and quantify the diffusion coefficient of nanoparticles through a polymer matrix, following polymyxin B treatment^59^. In contrast to MPT, which requires considerable specialized data analysis, the Transwell diffusion model offers a simple and fast method to analyze antibiotic diffusion.

## Conclusions

The mucin component in AS medium has a significant effect on polypeptide antibiotic diffusion and thus, antimicrobial activity. OligoG, in combination with colistin, markedly improved antibiotic penetration in this model, which correlated with increased antimicrobial efficacy against the *P. aeruginosa* CF isolate NH57388A.

## Materials and methods

### Materials

Alginate oligosaccharide*,* OligoG CF-5/20 was provided by AlgiPharma AS (Sandvika, Norway). 9-Fluorenylmethyl chloroformate (FMOC-Cl; purity ≥ 99.0%), trifluoroacetic acid (TFA; purity ≥ 99.0%), acetone (ACE; purity ≥ 99.5%), tetrahydrofuran (THF; purity ≥ 99.5%), boric acid (BA; purity ≥ 99.5%), sodium bicarbonate (SB; purity > 99.5%), colistin sulphate and polymyxin B were from Sigma-Aldrich (Poole, UK). Acetonitrile (ACN; purity ≥ 99.8%) was from Acros Organics (Geel, Belgium) and methanol (MeOH; purity ≥ 99.9%) from Fisher Scientific (Loughborough, UK). The LIVE/DEAD Baclight Bacterial Viability kit was from Invitrogen Molecular Probes (Paisley, UK) and BacTiter-Glo microbial cell viability kit from Promega (Southampton, UK). Sterile Transwell plates (0.4 μm pore polycarbonate membrane) were from Corning Inc. (New York, USA) and 24-well flat glass bottom black plates were from Greiner Bio-One (Stonehouse, UK). All chemicals and reagents were of analytical grade and used without any further purification. Highly purified Milli-Q water (MQ H_2_O) was produced using a PURELAB water purification equipment (Elga, High Wycombe, UK) and used in the preparation of all solutions (unless stated).

### Bacterial culture

All culture media was from LabM (Bury, UK). CF clinical isolate, *P. aeruginosa* NH57388A^60^ was subcultured on blood agar plates supplemented with 5% v/v defibrinated horse blood. Overnight cultures were grown in tryptone soy broth (TSB), and Mueller–Hinton broth (MHB) was used for biofilm growth.

### Artificial sputum (AS) medium

All constituents were obtained from Sigma-Aldrich (Poole, UK). Mucin (II) from porcine stomach (4% w/v) and DNA from salmon fish sperm (0.4% w/v) were dissolved in distilled water (dH_2_O) by stirring overnight at 4 °C. The remaining components: RPMI 1640 medium (2% v/v), egg yolk emulsion (0.5% v/v), sodium chloride (0.5% w/v), potassium chloride (0.22% w/v) and diethylenetriaminepentaacetic acid (0.0006% w/v) were then added with mixing until dissolved and the pH adjusted to 7.0 by drop-wise addition of sodium hydroxide (1 M). The AS medium was sterilized by *γ-*irradiation (26 Gy) and checked for sterility before use.

### The transwell diffusion model

The Transwell diffusion model consists of upper (donor) and lower (acceptor) compartments separated by a 0.4 µm pore microporous polycarbonate membrane. The model was developed to measure penetration of the antibiotic colistin through OligoG-treated AS medium and evaluate its effects on disruption of the *P. aeruginosa* NH57388A biofilm in the lower Transwell well.

Firstly, 90 µl of AS medium (untreated or pre-incubated with OligoG at 0.5, 1 and 2% w/v for 4 h) was added to the 6.5 mm Transwell upper donor well, while 600 µl of phosphate-buffered saline (PBS) was placed into the lower acceptor well. After 15 min, 10 µl of colistin (40 µg/ml in total system volume) was added to the upper donor well containing pre-treated AS. For surface treated samples, 10 µl of colistin (40 µg/ml) ± 1% w/v OligoG was added on the surface of the untreated AS medium. The plate was incubated statically at 37 °C for 48 h. Samples were collected from the lower acceptor well (at 2, 4, 6, 24 and 48 h) and stored at − 20 °C prior to analysis.

### Transwell sample pre-treatment for colistin quantification

The colistin detection method was adapted from previous studies^54^ but originally developed by Li et al.^61,62^. Colistin standards (0.25, 0.5, 1, 2 and 4 µg/ml in PBS) or samples collected from the Transwell diffusion model (15 µl) were mixed with 135 µl of internal standard (IS) solution (polymyxin B; 4 µg/ml in PBS) and precipitated by the addition of 150 µl ACN containing 0.1% v/v TFA, vortex-mixed for 10 s and centrifuged at 10,000 × *g* for 10 min. The supernatants (250 µl) were transferred to new polypropylene tubes and subjected to derivatization.

### Solid phase extraction (SPE) derivatization

SPE derivatization of colistin with fluorescent FMOC-Cl was performed on a 12-port vacuum manifold from Phenomenex (Macclesfield, UK). Briefly, the SPE C18-E cartridges (1 ml, 100 mg, 55 µm particle size, 70 Å pore size; Phenomenex, Macclesfield, UK) were conditioned sequentially with 1 ml ACE, MeOH and 1% w/w carbonate buffer (pH 10). After loading 200 µl of pre-treated sample, the cartridges were washed with 1 ml of 1% w/w carbonate buffer (pH 10) and then dried. FMOC-Cl (50 µl, 100 mM in ACN) was added to the top of the packed bed materials and allowed to react for 10 min. Then, the cartridges were dried under pressure for 5 min and washed with 1 ml of 95% v/v MeOH. Reaction derivatives were eluted using 900 µl ACE. To this, 600 µl of 0.2 M BA followed by 500 µl of ACN were added. The final solutions were filtered through a 0.45 µm polytetrafluoroethylene filter (13 mm in diameter, Millex; Millipore, Watford, UK) and stored at − 20 °C prior to analysis.

### High-performance liquid chromatography-fluorescence detection (HPLC-FLD)

Chromatographic analysis was performed using a Dionex ICS-3000 ion chromatography system (Thermo Scientific, Gloucester, UK) equipped with a Dionex RF-2000 fluorescence detector and a Dionex AS autosampler. Data was collected and processed using Chromeleon 6.8 software. Separation was achieved on an XSelect CSH C18 column (130 Å, 3.5 µm, 3.0 × 150 mm) connected to an XSelect guard column (130 Å, 3.5 µm, 2.1 × 5 mm) held at 30 °C with a flow rate of 0.8 ml/min (Waters; Wilmslow, UK). Samples were kept in the autosampler at 4 °C and eluted (30 µl) using a mixture of ACN, THF and MQ H_2_O (82:2:16 v/v/v) as a mobile phase over 20 min. The eluted peaks were recorded at excitation and emission wavelengths of 260 nm and 315 nm, respectively, with the gain set at 16 and response at 0.5. Colistin concentration in the lower Transwell compartment after diffusion through AS medium for 48 h was calculated as follows and expressed as mean ± SD (n = 3):

$${\text{Colistin }}\left( {\mu {\text{g}}/{\text{ml}}} \right) \, = { 1}/{\text{Cr}}_{{{\text{A}}/{\text{IS}}}} *{\text{ I}}\left( {\text{A}} \right)/{\text{I}}\left( {{\text{IS}}} \right) \, *{\text{ Q}}\left( {{\text{IS}}} \right)$$

where A = analyte, IS = internal standard, Cr = slope of the calibration curve, I = area under the curve and Q = quantity of IS in the sample.

A colistin calibration curve was generated using a linear regression method, by plotting the ratio of colistin (B and A) to IS (B2 and B1) using their summed areas under the curve versus the concentration ratio of colistin to IS (µg/ml).

### The *P. aeruginosa* NH57388A transwell biofilm diffusion model

Overnight cultures of *P. aeruginosa* NH57388A were adjusted to 10^7^ colony forming units (CFU)/ml in MHB. Then, 600 µl of inoculum was added to each of the wells of a 24-well microtitre plate and incubated statically at 37 °C for 24 h to allow biofilm growth. The biofilms were then rinsed once with PBS (600 µl) and the aqueous phase replaced with fresh MHB (600 µl). The 6.5 mm Transwell inserts were placed in each well and 90 µl of AS medium was added to the upper donor wells. After 15 min, 10 µl of colistin at 20, 40 and 60 µg/ml (in total system volume) ± 1% w/v OligoG was added to the donor wells and incubated statically at 37 °C for 24 h.

### *P. aeruginosa* NH57388A cell viability in the transwell biofilm diffusion model

Biofilms were grown in the Transwell model as described above. AS medium was subjected to OligoG (1% w/v) as a surface treatment or pre-incubated with OligoG (at 0.5, 1 and 2% w/v) for 4 h alongside appropriate dH_2_O controls. Transwell inserts were then carefully removed from the plate and the biofilm supernatants (containing planktonic cells) were collected before and after washing with PBS (600 µl). The remaining biofilm cells on the bottom of the 24-well plate were submerged in 600 µl of PBS and sonicated for 30 min to dislodge the attached biofilm cells. An ATP (luminescence) assay was then performed using a BacTiter-Glo microbial cell viability kit (according to the manufacturer’s instructions) to determine cell viability, measured on a Fluostar Omega microplate reader. The results were presented as percentage cell viability compared to untreated control cells and expressed as mean ± standard deviation (SD; n = 2).

### Biofilm disruption assay using the Transwell biofilm diffusion model

An adjusted *P. aeruginosa* NH57388A inoculum (50 µl) was placed in a Greiner glass-bottom 24-well black plate and allowed to adhere for 1 h. Then, 550 µl of MHB was added to the wells and biofilms were grown as described above. Transwell inserts and supernatants were carefully removed from the plate and then biofilms were incubated with 200 µl of LIVE/DEAD stain (0.8% v/v in PBS) in the dark for 20 min. Wells were rinsed once with PBS (600 µl), before addition of PBS (200 µl) to prevent dehydration prior to imaging. CLSM was performed using a Leica SP5 confocal microscope with × 63 magnification (under oil) and a step size of 0.79 µm. Dead/Live cell ratio was quantified using COMSTAT image analysis software^63^ and results were expressed as mean ± standard error of the mean (SEM; n = 15).

### Cell density of *P. aeruginosa* NH57388A in a transwell diffusion model

Biofilms were grown in the Transwell model as described above. Changes in cell density (absorbance at 600 nm) were recorded 24 h after addition of colistin ± 1% w/v OligoG onto the AS medium surface. Results were expressed as mean ± SD (n = 5).

### Statistical analysis

GraphPad Prism (version 9.2.0, 2021; San Diego, CA, USA) was used for statistical analysis. Statistical significance was indicated by *, where *p < 0.05. Analysis of variance (ANOVA) and Dunnett’s post hoc test were used to evaluate multiple group comparisons. Student’s unpaired t-test was used to determine the significance between the two independent groups.
